# Benchmarking Ionization Potentials from pCCD Tailored Coupled Cluster Models

**DOI:** 10.1021/acs.jctc.4c00172

**Published:** 2024-05-16

**Authors:** Marta Gałyńska, Katharina Boguslawski

**Affiliations:** Institute of Physics, Faculty of Physics, Astronomy, and Informatics, Nicolaus Copernicus University in Toruń, Grudziadzka 5, 87-100 Toruń, Poland

## Abstract

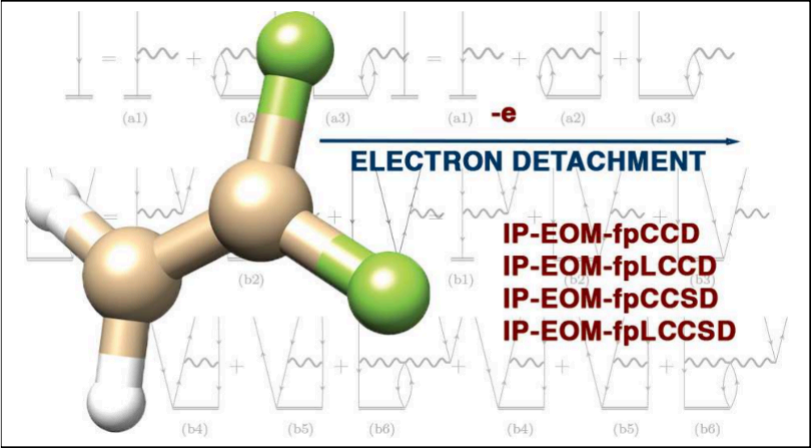

The ionization potential (IP) is an important parameter providing essential insights into the reactivity of chemical systems. IPs are also crucial for designing, optimizing, and understanding the functionality of modern technological devices. We recently showed that limiting the CC ansatz to the seniority-zero sector proves insufficient in predicting reliable and accurate ionization potentials within an IP equation-of-motion coupled-cluster formalism. Specifically, the absence of dynamical correlation in the seniority-zero pair coupled cluster doubles (pCCD) model led to unacceptably significant errors of approximately 1.5 eV. In this work, we aim to explore the impact of dynamical correlation and the choice of the molecular orbital basis (canonical vs localized) in CC-type methods targeting 230 ionized states in 70 molecules, comprising small organic molecules, medium-sized organic acceptors, and nucleobases. We focus on pCCD-based approaches as well as the conventional IP-EOM-CCD and IP-EOM-CCSD. Their performance is compared to the CCSD(T) or CCSDT equivalent and experimental reference data. Our statistical analysis reveals that all investigated frozen-pair coupled cluster methods exhibit similar performance, with differences in errors typically within chemical accuracy (1 kcal/mol or 0.05 eV). Notably, the effect of the molecular orbital basis, such as canonical Hartree–Fock or natural pCCD-optimized orbitals, on the IPs is marginal if dynamical correlation is accounted for. Our study suggests that triple excitations are crucial in achieving chemical accuracy in IPs when modeling electron detachment processes with pCCD-based methods.

## 1 Introduction

The reliable determination of ionization potentials (IP) is crucial for the theoretical modeling of molecular electronic structures and molecular properties. The IP provides information about the system’s reactivity as it facilitates measuring the strength of one electron being attached to the molecular bulk and quantifying the molecule’s ability to form a more positively charged ion. This information can be further utilized to design, optimize, and comprehensively understand the functionality of modern technological devices such as photovoltaic (PV) cells, light-emitting diodes, or sensors.^1,2^ For instance, a critical factor in designing novel organic-based donor and acceptor molecules in organic PV devices^1^ is the knowledge of the energies of the highest occupied molecular orbital (HOMO) and the lowest unoccupied molecular orbital (LUMO) and the corresponding HOMO–LUMO gap. From a theoretical perspective, one of the simplest approximations to deduce orbital energies exploits the diagonal elements of the Fock matrix and the electron repulsion energy.^3^ More reliable orbital energies can be, for instance, obtained from the ionization potential (IP)^4−7^ and electron affinity (EA)^8^ variants of equation-of-motion (EOM)^9−11^ applied on top of a coupled cluster reference wave function. The resulting IPs and EAs are then exploited to predict the so-called charge gap.

Apart from CC approaches,^12−14^ various calculation protocols have been proposed to investigate the electron detachment process, including density functional theory and its time-dependent formulation,^15,16^ configuration interaction models,^17,18^ perturbation theory,^19^ algebraic-diagrammatic construction (ADC) schemes,^20−23^ and Monte Carlo methods.^24,25^ These methods feature a diverse spectrum related to their accuracy and computational complexity, cost, and resource requirements. Furthermore, they can be applied to a wide range of chemical compounds, varying in complexity and size. Among these methods, different variants of IP-EOM-CC^6,7,26−28^ have become well-established correlation-based methods, mainly employed for simulating photoelectron spectroscopy.^8,10,29−40^

The most common CC ansatz is constrained to single and double excitations (CCSD) but can be rather easily extended to perturbatively account for triple substitutions (CCSD(T)), which is commonly known as the gold standard of quantum chemistry. Those, as well as further extensions of the CC ansatz, including full triples (CCSDT), quadruples, and higher excitations, can be combined with an IP-EOM formalism to describe ionized states.^32^ Although those EOM-CC methods^11,33,41^ are highly reliable in terms of accuracy, they are remarkably expensive and hence limited to relatively small system sizes. Thus, a significant effort has been made to devise alternatives of similar accuracy but more reasonable computational complexity. The simplified IP-EOM pair coupled cluster doubles (IP-EOM-pCCD) variant^42^ proved an inexpensive alternative to model open-shell electronic structures within the pCCD^43−45^ model. pCCD was originally introduced as a geminal-based wave function^46^ ansatz using two-electron functions as building blocks for the electronic wave function.^46−63^ Other examples are strictly localized geminals,^64−66^ the antisymmetrized product of strongly orthogonal geminals,^47,50,53,67^ and the generalized valence bond perfect pairing^68,69^ models, to name a few. Such models are a promising alternative to conventional electronic structure approaches, which are typically constructed from one-electron functions.

Initial numerical studies^42^ demonstrated that the accuracy of IP-EOM-pCCD approaches closely matches the accuracy of CCSD(T) or the density matrix renormalization group^70−73^ (DMRG) algorithm in open-shell electronic structures. We recently presented a benchmark study to assess the accuracy of the IP-EOM-pCCD method in predicting ionization energies.^74^ In ref (74), we compared the vertical ionization energies obtained in the space of one-hole (1h) and two-hole-one-particle (2h1p) states for three types of molecular orbitals (canonical Hartree–Fock, Pipek-Mezey localized, and natural pCCD orbitals). Our study suggests that the orbital-optimized IP-EOM-pCCD method, restricted to the 2h1p operator, demonstrated the highest accuracy among the investigated methods. However, due to the absence of dynamical correlation, we observed unacceptably large errors in IPs of approximately 1.5 eV.

As demonstrated previously,^75,76^ natural pCCD orbitals present a promising alternative to canonical Hartree–Fock orbitals, serving as a reference wave function for more sophisticated calculations. Consequently, the question arises whether it is possible to achieve an accuracy comparable to more elaborate approaches (like CCSDT or higher) employing natural pCCD-optimized orbitals in combination with simplified CC ansätze, which account for dynamical correlation. Thus, in the current work, we explore, for the first time, both the effect of dynamical correlation and the choice of the molecular orbital basis (canonical vs localized) in CC-type methods including up to double excitations. Specifically, we focus on various pCCD-tailored CC flavours^76−78^ and compare their performance to the conventional CCSD method exploiting a canonical and pCCD-optimized localized molecular orbital basis. Specifically, we investigate the influence of dynamical correlation to the IP values determined by six different approaches using the natural pCCD-optimized orbitals, namely frozen-pair (fp)CC^77^ methods (IP-EOM-fpCCD and IP-EOM-fpCCSD), their linearized (fpLCC)^78^ variants (IP-EOM-fpLCCD and IP-EOM-fpLCCSD), and conventional IP-EOM-CCD and IP-EOM-CCSD,^4−7^ and compare their performance to the CCSD(T) or CCSDT equivalents and experimental reference data. Our test sets contain small organic molecules, medium-sized organic acceptors, and nucleobases. Specifically, the acceptor set contains chemical families typically encountered in organic electronics, like acenes, nitro compounds, nitriles, quinones, and anhydrides. Nucleobases, on the other hand, are commonly studied to anticipate the accurate description of charge-transfer states in polynucleotides or to elucidate phenomena related to photodamage or repair of genetic material.^79−81^

This work is structured as follows: In Section 2, we briefly review the investigated theoretical models. Section 3 provides an overview of the computational methodology. Section 4 presents the numerical results, including a statistical analysis. Finally, we conclude in Section 5.

## 2 Theory

The pCCD^43−46^ ansatz is a simple reduction of the single-reference CCD approach, where the cluster operator only contains electron-pair excitations ,
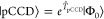
1and
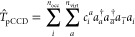
2where |Φ_0_⟩ is some reference determinant,  are the elementary annihilation (creation) operators for α (*p*) and  electrons, and *c*_*i*_^*a*^ are the pCCD cluster amplitudes. The above sum runs over all occupied *i* and virtual *a* orbitals. Typically, the pCCD molecular orbitals are optimized,^44,45,82,83^ which re-establishes size consistency and yields localized and symmetry-broken orbitals that allow us to simulate quantum states with (quasi-)degeneracies.^84^ Numerical examples comprise bond-breaking processes in small molecules,^55,76,85−90^ heavy-element-containing compounds featuring lanthanide^91^ or actinide^86,90,92−97^ atoms, organic electronics,^75,98^ and electronically excited states.^42,93−95,99−101^ Although these numerical studies support pCCD to be a promising alternative to capture static/nondynamic electron correlation effects,^102−104^ a large fraction of the correlation energy cannot be captured by electron-pair states alone. This missing correlation energy is commonly attributed to so-called broken-pair states. These correlation effects are commonly included *a posteriori* using various state-of-the-art techniques.^46^

One possibility to account for dynamical correlation is to exploit a coupled cluster correction with a pCCD reference function.^76−78^ For instance, in the frozen-pair coupled cluster (fpCC) ansatz,^76,77^
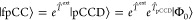
3the pCCD wave function is taken as the fixed reference function and the  cluster operator contains electron excitations beyond electron-pair excitations. Thus, the cluster operator of fpCCD is defined as , while the cluster operator of fpCCSD includes also single excitations, . We should stress that fpCC theory can be considered as a conventional tailored coupled cluster approach.^105−108^ The fpCC ansatz can be further simplified by truncating the Baker–Campbell–Hausdorff expansion after the second term (concerning all nonpair excitations) and hence including only linear terms in .^78,109^ Strictly speaking, the frozen-pair Linearized CC correction (fpLCC) does not fall into the category of tailored CC methods. Nonetheless, we will use the acronym fpLCC due to its simplicity (originally, fpLCC was introduced as pCCD-LCC). The wave function ansatz of fpLCC is approximated as
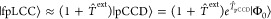
4

We should note that all disconnected terms containing  still appear in the fpLCC amplitude equations as the exponential ansatz of pCCD is not linearized. For instance, terms associated with  and  have to be considered in fpLCCD-type methods. Thus, in fpLCC, the coupled cluster equations are linear concerning nonpair amplitudes  but the coupling between all pair- and nonpair amplitudes is included.

Since we formally work in a single-reference CC picture, we can straightforwardly employ single-reference CC techniques to target, for instance, electronically excited, ionized, and electron-attached states.^8,39,110^ Another possible extension are spin-flip EOM-CC methods.^111^ Specifically, for ionized states, we can employ the IP-EOM formalism, where we use a linear ansatz on top of the closed-shell fp(L)CC reference function to parametrize the k-th (ionized) state
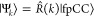
5where the operator *R̂*(*k*) generates the targeted ionized state *k* from the initial fp(L)CC reference state. In the single IP-EOM formalism, R̂(k) reads
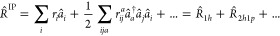
6where we introduced the hole (h, encoding ) and particle (p, encoding ) labels and dropped the k-dependence for better readability. The ionized states are then obtained by solving the corresponding EOM equations
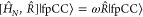
7where ω = Δ*E* – Δ*E*_0_ is the energy corresponding to the ionization process concerning the fpCC ground state, while  is the normal product form of the Hamiltonian. We can rewrite the above equation in the well-known form
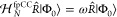
8where  is the similarity transformed Hamiltonian of the used fpCC flavor in its normal-product form
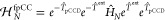
9

**Figure 1:**
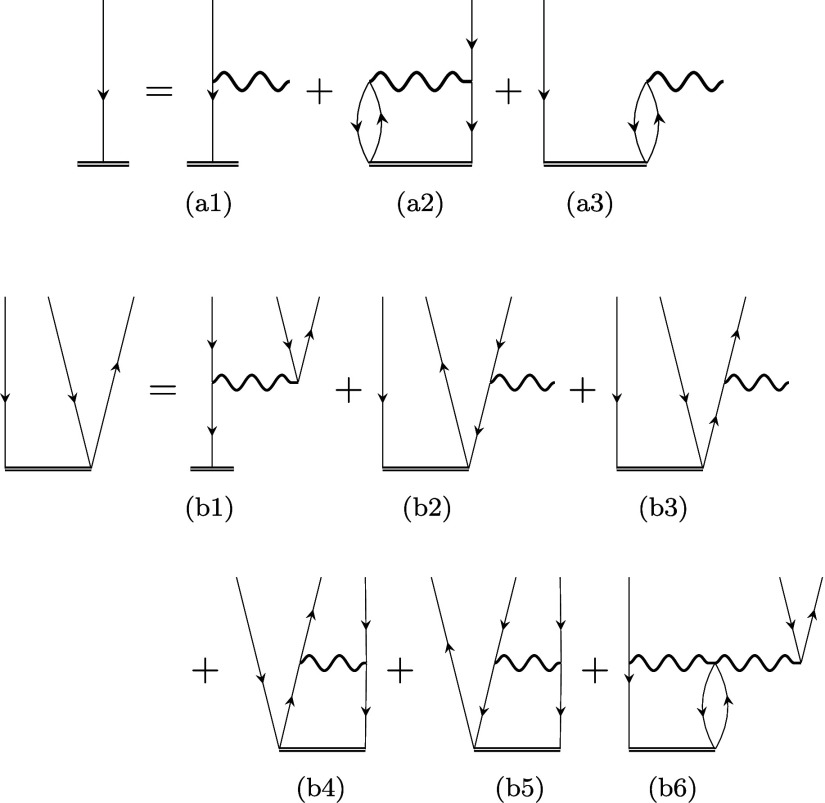
Diagrammatic representation of the IP-EOM-fp(L)CCSD equations (antisymmetrized formalism).

The ionization energies are obtained by iteratively diagonalizing  of the chosen pCCD-based CC correction. Note that depending on the selected CC model, the similarity transformed Hamiltonian either contains all nonvanishing nonlinear terms (here,  and ) or only nonlinear terms associated with the electron-pair excitation operator (here,  and ). The diagrammatic representation of the IP-EOM-fpCC equations is shown in Figure 1 (see also, for instance, ref (32) for the diagrammatic form and its algebraic expressions). We should note that the IP-EOM-fpCC(S)D equations are similar in form to the conventional IP-EOM-CCSD formalism. However, in the IP-EOM-fpLCCSD method, the effective Hamiltonian diagrams (a1), (b1), (b2), (b3), (b4), (b5) do not contain the disconnected  terms, while, in addition, diagram (b1) lacks the  part. Furthermore, the  term contained in (b1) is replaced by the simpler  counterpart due to the truncation of the BCH expansion of the LCC correction (see also eq 4). Finally, we should note that we focused on the *S*_*z*_ = −0.5 case^8,42^ in its spin-dependent and spin-summed versions. The spin-summed working equations can be obtained by either diagrammatic or algebraic spin summation (see also ref (32)) of the IP-EOM-fp(L)CC equations shown in Figure 1. For the latter case, the targeted ionized (open-shell) states are the doublet states of the *S*_*z*_ = −0.5 spin block.

### 2.1 Notes on Scaling

The formal computational scaling of IP-EOM-pCCD-based methods is similar to the conventional IP-EOM-CCD/IP-EOM-CCSD flavors. While the evaluation of the amplitude equations (namely, the vector function) has a computational cost of , where *o* is the number of occupied and *v* the number of virtual orbitals, respectively, one matrix-vector multiplication of the effective Hamiltonian with a trial vector of a Davidson iteration step formally scales as  (using suitable intermediates). Compared to IP-ADC flavors,^112^ the computational cost of IP-EOM-pCCD-based methods is between the ADC(2) and ADC(3) counterparts. While the former features an overall scaling of  (amplitude evaluation), the overall cost increases to  for the latter. Finally, the matrix-vector multiplication for both IP-ADC(3) and IP-fp(L)CC methods scales similarly as , while it reduces to  for IP-ADC(2). Thus, all investigated IP-fp(L)CC models are of similar computational complexity as IP-ADC(3).

## 3 Computational Details

The vertical ionization potentials (IP) were calculated using different CCD- and CCSD-type flavors as implemented in a developer version of the PyBEST v1.4.0-dev0 software package.^113−115^ These included IP-EOM-fpCCD, IP-EOM-fpCCSD, IP-EOM-fpLCCD, IP-EOM-fpLCCSD, IP-EOM-CCD, and IP-EOM-CCSD. In all CC calculations (electronic ground states and IP-EOM), the pCCD-optimized orbitals (labeled as “(pCCD)”) were used to construct the reference determinant |Φ_0_⟩, which were obtained through a variational orbital-optimization protocol of the pCCD reference calculation.^44,45,82,83^ The optimization protocol used in pCCD calculations automatically selects the reference determinant according to the pCCD natural occupation numbers.

The ionization energies were computed in the space of two-hole-one-particle (2h1p) states as previous studies indicate that errors in IPs are significantly reduced compared to the use of only one-hole (1h) states.^74^ No symmetry constraints were applied to allow the algorithm to freely relax the orbitals resulting in a symmetry-broken, localized molecular orbital basis.

A frozen core was used in all CC calculations, keeping the 1s orbitals for C, N, O, and F and 1s, 2s, and 2p orbitals for Si, P, S, and Cl frozen. We should note that preliminary tests indicate a minimal impact on the IP values when freezing core orbitals.

**Figure 2:**
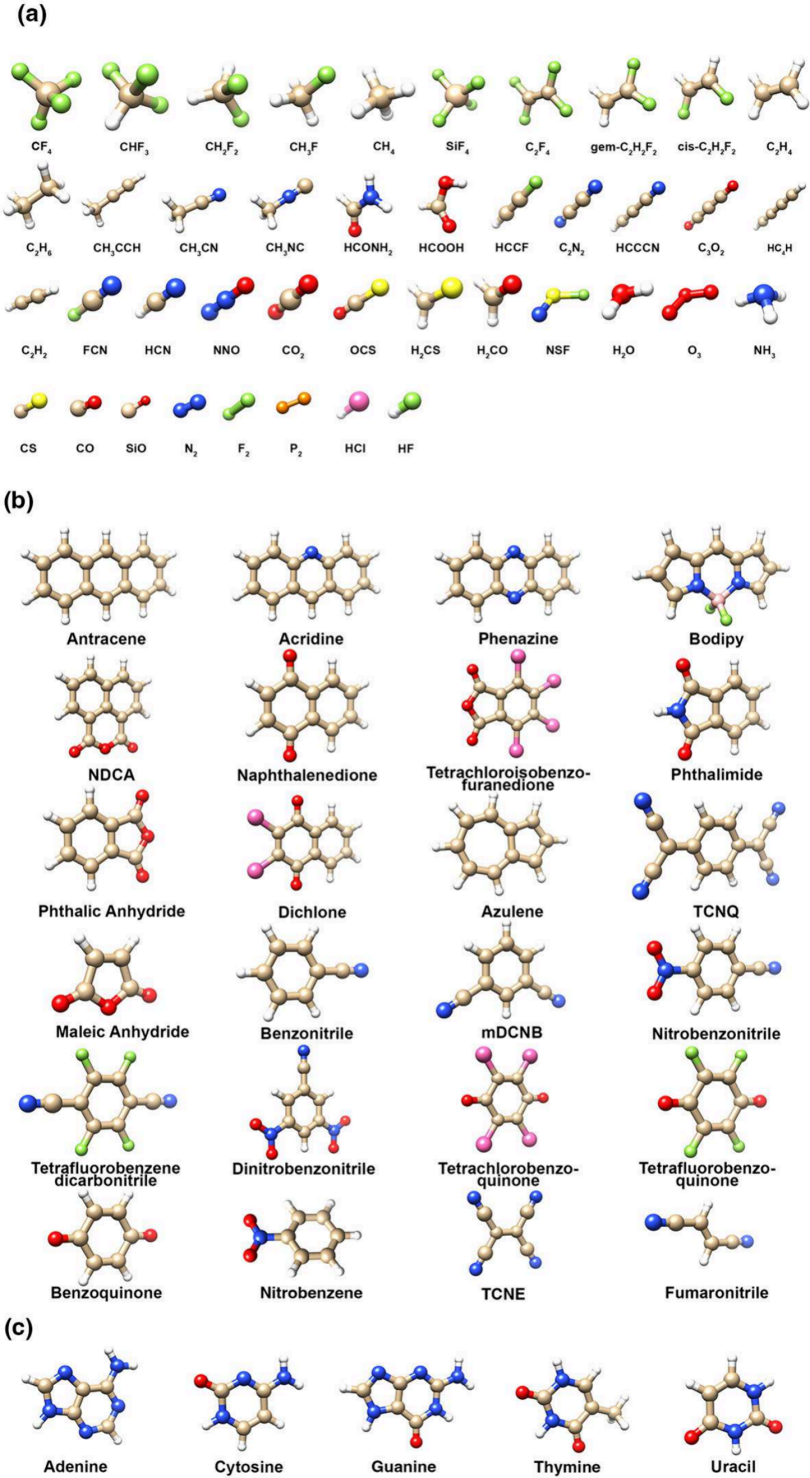
Three benchmark sets investigated in this work containing (a) 41 molecules relaxed at the CCSD(T)/aug-cc-pVTZ level provided in ref (117), (b) 24 organic molecules relaxed at the B3LYP/6-311G** level provided in ref (120), and (c) 5 nucleobases optimized using the B3LYP/6-31G(2df,p) method obtained from refs (127, 128).

Our test set includes three sets of molecules shown in Figure 2, namely (a) small organic molecules, (b) medium-sized organic acceptors, and (c) nucleobases. The first one contains 41 small molecules (Figure 2(a)), whose molecular geometries were relaxed using the CCSD(T)/aug-cc-pVTZ method^116^ and are available in the Supporting Information of ref (117). In total, we optimized 201 IP states and compared the performance of our IP-EOM-CC methods to theoretical and experimental reference data. This series of calculations employed the cc-pVTZ basis set of Dunning,^118^ facilitating a direct comparison with previously published vertical IPs obtained using the IP-EOM-CCSDT model,^117^ various forms of the unitary coupled-cluster (IP-EOM-UCC) approach, and algebraic-diagrammatic construction (IP-EOM-ADC) methods.^119^

The second set (Figure 2(b)) includes 24 organic molecules relaxed with B3LYP/6-311G**, whose molecular structures are available in the Supporting Information of ref (120). In all calculations, Dunning’s aug-cc-pVDZ^118,121−123^ basis set was used to allow for a direct comparison of our pCCD-based first ionization energies to those obtained by conventional methods like CCSD (exploiting canonical Hartree–Fock orbitals), different variants of algebraic-diagrammatic construction (ADC(2), SCS-ADC(2), and SOS-ADC(2)) schemes, and an approximate coupled cluster singles and doubles model (CC2)^124^ against CCSD(T)/aug-cc-pVDZ^125^ reference data and experimental findings.^126^

The third set (Figure 2(c)) includes 5 nucleic acid bases (guanine, adenine, cytosine, thymine, and uracil) relaxed previously at the B3LYP/6-31G(2df,p) level.^127,128^ For this last set, we followed ref (124) and calculated the first vertical ionization energies for the cc-pVDZ and cc-pVTZ basis sets and extrapolated them to the complete basis set (CBS) limit. For that purpose, we applied a two-point procedure as in refs (129, 130), where the asymptotic value of the total electronic energies in the CBS limit is extrapolated using the following fitting function
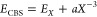
10

In the above equation, *X* is the cardinal number of the atomic basis set (*X* = 2 for the cc-pVDZ basis, *X* = 3 for the cc-pVTZ basis, etc.), *E*_*X*_ is the corresponding total energy of a specific state, and *a* is some fitting parameter. In this work, we approximated the CBS limit for *X* = 2, 3 using the same collection of methods as for the second test set of organic acceptors.

## 4 Results and Discussion

**Figure 3:**
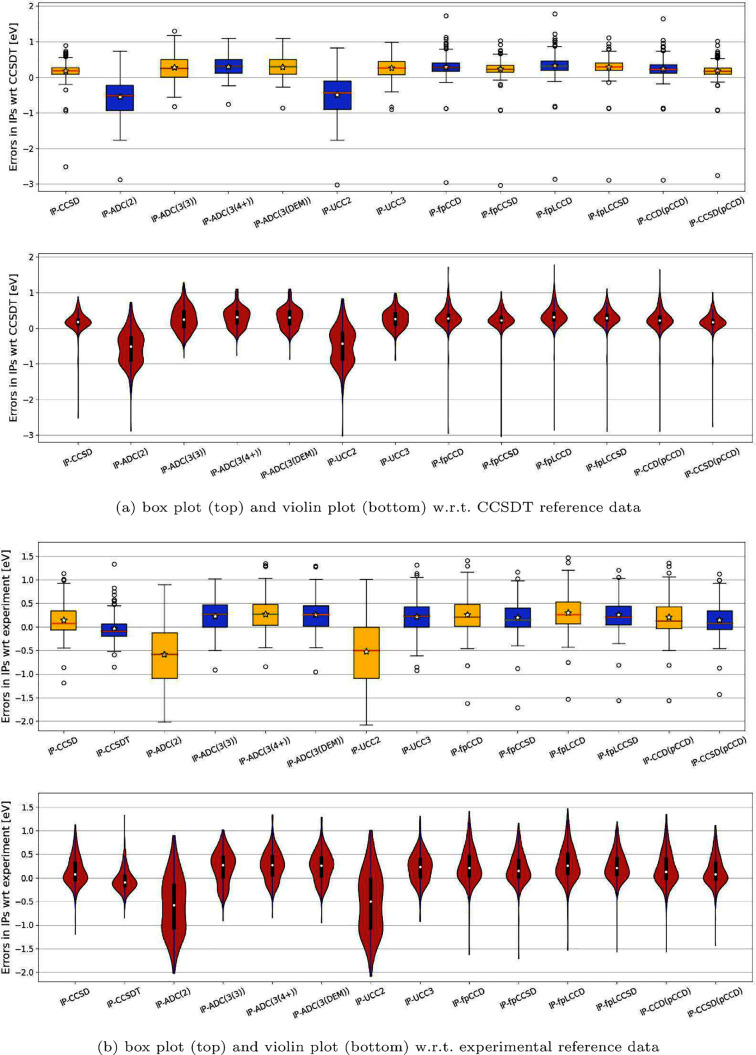
Box plots presented at the top and violin plots at the bottom, illustrating errors [eV] derived from selected methods (refer to Table 1 for numerical values) for the first test set of small organic molecules. All errors are reported relative to either (a) IP-EOM-CCSDT or (b) experimental reference data. For brevity, we have omitted the EOM prefix in IP-EOM-CC-type methods. A star in each box plot denotes the mean value, while a white dot in each violin plot represents the median value.

Table 1 summarizes our statistical analysis, including mean errors (ME), mean absolute errors (MAE), and root-mean-square errors (RMSE) calculated using IP-EOM-fpCCD, IP-EOM-fpCCSD, IP-EOM-fpLCCD, IP-EOM-fpLCCSD, conventional IP-EOM-CCD(pCCD), and IP-EOM-CCSD(pCCD) of 201 ionized states in 41 molecules shown in Figure 2(a). The footnote in Table 1 defines the error measures used in this work. We should note that the labels CCD(pCCD) and CCSD(pCCD) indicate that the corresponding CCD and CCSD calculations were done employing pCCD-optimized natural orbitals (or equivalently the orbital-optimized pCCD reference determinant in the CC ansatz). The upper section of Table 1 presents error values concerning IP-EOM-CCSDT reference data,^117^ while the lower section reports the corresponding errors relative to experimental results.^117^ For a direct comparison, the table includes data from IP-EOM-CCSD calculated with a canonical Hartree–Fock reference determinant (or molecular orbital basis) indicated as IP-CCSD(HF), two variations of the unitary coupled cluster ansatz (IP-UCC2 and IP-UCC3), and four variants of non-Dyson algebraic diagrammatic construction schemes (ADC(2), ADC(3(3)), ADC(3(4+)), and ADC(3(DEM)).^119^ Furthermore, Figure 3(a) visualizes, using box and violin plots, the locality, spread, skewness, and distribution of errors of all targeted ionization potentials concerning IP-EOM-CCSDT. Figure 3(b) displays an equivalent analysis for experimental data. The individual ionization energies obtained by all methods investigated in this work are accessible in the Supporting Information (SI).

**Table 1:** Statistical Error Measures [eV], Such as Mean Error (ME), Mean Absolute Error (MAE), Root-Mean-Square Error (RMSE), and Mean Percentage Error (MPE) for the First Set of Molecules Shown in Figure 2(a)^a^

	Errors w.r.t. IP-EOM-CCSDT			
Method	ME	MAE	RMSE	MPE
IP-fpCCD	0.295	0.346	0.461	2.0
IP-fpLCCD	0.335	0.382	0.489	2.3
IP-CCD(pCCD)	0.241	0.297	0.418	1.7
IP-fpCCSD	0.239	0.293	0.399	1.7
IP-fpLCCSD	0.293	0.341	0.432	2.0
IP-CCSD(pCCD)	0.183	0.238	0.350	1.3
IP-pCCD^74^	–1.535	1.535	1.633	9.8
IP-CCSD(HF)^117^	0.186	0.241	0.341	1.4
IP-UCC2^119^	–0.488	0.579	0.728	3.6
IP-UCC3^119^	0.260	0.306	0.377	1.8
IP-ADC(2)^119^	–0.545	0.607	0.737	3.8
IP-ADC(3(3))^119^	0.269	0.351	0.442	2.1
IP-ADC(3(4+))^119^	0.306	0.339	0.418	2.0
IP-ADC(3(DEM))^119^	0.292	0.334	0.411	2.0

	Errors w.r.t. experiment			
Method	ME	MAE	RMSE	MPE
IP-fpCCD	0.259	0.336	0.457	1.9
IP-fpLCCD	0.299	0.361	0.481	2.1
IP-CCD(pCCD)	0.206	0.303	0.422	1.7
IP-fpCCSD	0.203	0.283	0.391	1.6
IP-fpLCCSD	0.256	0.311	0.415	1.8
IP-CCSD(pCCD)	0.147	0.253	0.358	1.5
IP-pCCD^74^	–1.570	1.570	1.697	10.0
IP-CCSD(HF)^117^	0.150	0.252	0.351	1.4
IP-CCSDT^117^	–0.035	0.197	0.262	1.2
IP-UCC2^119^	–0.523	0.684	0.841	4.2
IP-UCC3^119^	0.225	0.312	0.394	1.8
IP-ADC(2)^119^	–0.580	0.693	0.844	4.3
IP-ADC(3(3))^119^	0.233	0.356	0.419	2.1
IP-ADC(3(4+))^119^	0.271	0.342	0.423	2.0
IP-ADC(3(DEM))^119^	0.256	0.339	0.419	2.0

a Assessed based on the ionization potentials (IP) calculated using various methods: IP-EOM-fpCCD, IP-EOM-fpCCSD, IP-EOM-fpLCCD, IP-EOM-fpLCCSD, IP-EOM-CCD(pCCD), and IP-EOM-CCSD(pCCD), listed in the upper part of each block, are derived in this work. All these CC approaches exploit natural pCCD-optimized orbitals (or the orbital-optimized pCCD reference determinant) and are conducted in the space of two-hole-one-particle (2h1p) states. The corresponding errors in IPs for CCSD done with Hartree-Fock orbitals, unitary coupled cluster (UCC), algebraic-diagrammatic construction (ADC) methods, and pCCD, using natural pCCD-optimized orbitals, are presented in the lower part of each block. The errors in IPs were calculated concerning IP-EOM-CCSDT^117^ (top) and experimental data^117^ (bottom). The definitions for ME, MAE, RMSE, and MPE are ME = , MAE = , RMSE = , MPE =

The effect of adding dynamical correlation, that is including the seniority two and seniority four sectors in the fpCC reference function of the IP-EOM-fpCCD method, provides a considerable improvement of the ionization energies in comparison to the seniority zero orbital-optimized pCCD method. Specifically, the MAE and RMSE errors are reduced by approximately 1.2 eV on average if we go beyond the seniority-zero sector in the CC reference function. This supports the original finding^74^ that dynamical correlation is needed to correctly describe the electron detachment process within pCCD-based methods. Using a simplified version of the frozen-pair methods, IP-EOM-fpLCCD increases slightly the ME, MAE, and RSME errors compared to IP-EOM-fpCCD by 0.040 (0.040) eV, 0.036 (0.025) eV, 0.028 (0.024) eV, respectively, with respect to IP-EOM-CCSDT reference data (experiment). On the other hand, IP-EOM-CCD(pCCD) (conventional IP-EOM-CCD with an orbital-optimized pCCD reference determinant) decreases the errors of IP-EOM-fpCCD by 0.054 (0.053) eV for ME, 0.049 (0.033) eV for MEA, are 0.043 (0.035) eV for RMSE with respect to IP-EOM-CCSDT (experimental) results.

A similar trend is observed for CCSD-based approaches. Specifically, IP-EOM-fpLCCSD yields the largest errors, while IP-EOM-CCSD(pCCD) (conventional IP-EOM-CCSD with an orbital-optimized pCCD reference determinant) exhibits the smallest errors compared to theoretical (experimental) reference values. Nonetheless, their differences in errors are acceptable, amounting up to around 0.11 eV (or 2.5 kcal/mol). Most importantly, including single excitations slightly reduces the ME, MAE, and RMSE values by 0.041 to 0.061 eV between the frozen-pair CCD and CCSD methods, respectively. Regarding statistical errors, IP-EOM-fpCCSD is the most accurate among all investigated pCCD-based approaches investigated in this work resulting in an MPE of only 1.7% (1.6%) concerning CCSDT (experimental) reference data. Specifically, it yields very similar errors to the IP-EOM-UCC3 method identified as the best among recently investigated approximations.^117^ Concerning IP-EOM-CCSDT (experiment), the relative error measures between IP-EOM-fpCCSD and IP-EOM-UCC3 are ΔΔME = 0.021 (0.022) eV, ΔΔMAE = 0.013 (0.029) eV, and ΔΔRMSE = −0.022 (0.003) eV, where ΔΔ *i*ndicates the difference between the IP-EOM-fpCCSD and IP-EOM-UCC3 error measures.

Figure 3 displays the corresponding box and violin plots of all errors in IPs. Specifically, box and violin plots are useful tools for representing the locality, spread, skewness, and distribution of large sets of values, such as errors. In the box plots of Figure 3, the yellow and blue boxes represent the interquartile range, containing 50% of the errors. The whiskers extend to show the remaining range of errors (the total range of scope). The red lines represent the median values, the white stars indicate the mean, and the circles show outliers. The skewness of errors measures the asymmetry of the distribution resulting in a (left or right) data tail. A positive (right) skewness indicates that the tail of the errors extends above the median, featuring a mean larger than the median value. Conversely, a negative (left) skewness features a tail of the errors extending below the median, with a mean smaller than the median value. In addition to a statistical summary of mean/median and interquartile ranges, violin plots provide the full distribution of the data. In the violin plots of Figure 3, the white circles represent the median value, and the bold black line represents the interquartile range.

Our box plots (see Figure 3 top) illustrate that the differences in errors among all fpCC methods are very similar, displaying an almost identical dispersion of 50% of errors (highlighted in yellow and blue boxes). The total range of scope (indicated by the whiskers) diminishes slightly with the addition of single excitations. However, the differences are minimal. The violin plots (see Figure 3 bottom) highlight interquartile ranges distributed closely around the median. In all cases, the skewness of errors is nearly zero (symmetric distributions) when outliers are excluded, in particular when plotting against CCSDT reference values. For the experimental reference data, the tails of the errors are visibly shifted upward, indicating right skewness. Although the dispersion of results is slightly smaller when using the IP-EOM-CCSDT method as a reference, a similar trend of error dispersion can be seen for both references (theoretical and experimental). All frozen-pair variants exhibited an accuracy range similar to IP-EOM-CCSD conducted with canonical HF molecular orbitals, demonstrating a comparable dispersion and skewness of errors. Compared to other approximate methods (ADC and UCC), the proposed pCCD-based IP-EOM variants predict more compact and symmetric distributions of errors. Specifically, IP-EOM-ACD(2) and IP-EOM-UCC2 provide symmetric, but broad and dumbbell-shaped error distributions with the median being shifted downward (underestimation of IPs). Increasing the computational complexity in ADC and UCC shifts the distribution upward (overestimation of IPs). While all IP-EOM-ADC(3) flavors and IP-EOM-UCC3 feature similar median as the investigated fpCC variants, the corresponding error distributions are broadened, asymmetric, and egg-shaped. Despite similar computational costs, IP-EOM-fpCC methods outperform the investigated IP-EOM-ADC(3) models and the IP-EOM-UCC3 approach.

Finally, the IP-EOM-CCSD results reported by Ranasinghe et al.^117^ allow us to directly assess the effect of the choice of the molecular orbital basis on molecular properties, that is if the performance is significantly different between canonical HF and natural pCCD-optimized orbitals. Surprisingly, the errors are almost identical and exhibit resilience to the choice of the reference wave function. Furthermore, the overall appearance of error distribution, skewness, and even the positioning of outliers presented in the box and violin plots in Figure 3 is almost identical in both cases (differences lie within chemical accuracy). This suggests that including triple excitations in the theoretical model will be crucial for further improving the accuracy of pCCD-based approaches in predicting IPs.

The ME, MAE, RMSE, and MPE errors calculated for the second test set containing 24 organic acceptor molecules are listed in Table 2. Despite the smaller size of this set, the errors exhibit similar trends as those observed for the first set (small organic molecules summarized in Figure 2(a). The IP-EOM-fpLCCD variant consistently results in the largest errors among all investigated CCD-type methods. Including nonlinear broken-pair excitations in the CC correction (IP-EOM-fpCCD) reduces the ME, MAE, and RMSE errors by approximately 0.1 eV. If all seniority-sectors are coupled (IP-EOM-CCD) the errors shrink by 0.1 to 0.2 eV. Similarly to the previous data set, including single excitations decreases the errors with respect to the corresponding CCD variant by 0.02 to 0.08 eV. As observed above, the IP-EOM-fpCCSD model appears to be the best among all investigated pCCD-based methods, showing an MPE of 1.2% (2.0%) relative to the CCSD(T) (experimental) reference data. This MPE is approximately halved if all seniority-sectors are treated on an equal footing (IP-EOM-CCD/IP-EOM-CCSD). In general, all frozen pair CC methods perform considerably better than the CC2 approximation and various flavors of ACD(2).^124^ While the performance of IP-EOM-fpLCCSD lies between IP-EOM-SCS-ADC(2) and IP-EOM-SOS-ADC(2), IP-EOM-fpCCSD lowers the MPE by a factor of 2 (IP-EOM-SOS-ADC(2)) or 3 (IP-EOM-SOS-ADC(2) and IP-EOM-ADC(2)), while MAE and RSME differ only by 0.1 eV between those two methodologies. Finally, the choice of the reference wave function (or the molecular orbital basis) does not affect the errors in IPs. Thus, treating the seniority-zero (pCCD) and seniority-two/four sectors (post-pCCD) successively deteriorates IPs compared to their composite optimization (CCD/CCSD).

**Table 2:** Statistical Error Measures [eV], Such as Mean Error (ME), Mean Absolute Error (MAE), Root-Mean-Square Error (RMSE), Mean Percentage Error (MPE), and Standard Deviation (SD) Calculated for the Second Set of Molecules (Figure 2(b)—Organic Acceptors)^a^

	Errors w.r.t. IP-EOM-CCSD(T)				
Method	ME	MAE	RMSE	MPE	SD
IP-pCCD	–2.002	2.002	2.017	21.1	0.253
IP-fpCCD	0.172	0.187	0.222	1.9	0.143
IP-fpLCCD	0.284	0.285	0.316	2.9	0.142
IP-CCD(pCCD)	0.092	0.125	0.155	1.3	0.127
IP-fpCCSD	0.097	0.120	0.140	1.2	0.103
IP-fpLCCSD	0.243	0.255	0.272	2.6	0.123
IP-CCSD(pCCD)	0.014	0.068	0.089	0.7	0.089
IP-CCSD(HF)^124^	0.008	0.070	0.089	0.7	0.090
IP-CC2^124^	–0.572	0.572	0.636	5.9	0.284
IP-ADC(2)^124^	–0.504	0.504	0.591	5.3	0.315
IP-SCS-ADC(2)^124^	–0.281	0.287	0.355	3.1	0.222
IP-SOS-ADC(2)^124^	–0.168	0.222	0.255	2.4	0.197

	Errors w.r.t. experiment				
Method	ME	MAE	RMSE	MPE	SD
IP-pCCD	–1.975	1.975	2.006	20.7	0.335
IP-fpCCD	0.180	0.253	0.324	2.5	0.258
IP-fpLCCD	0.288	0.346	0.390	3.5	0.251
IP-CCD(pCCD)	0.098	0.203	0.281	2.0	0.251
IP-fpCCSD	0.107	0.200	0.280	2.0	0.247
IP-fpLCCSD	0.249	0.330	0.363	3.4	0.252
IP-CCSD(pCCD)	0.019	0.166	0.257	1.7	0.245
IP-CCSD(HF)^124^	0.011	0.167	0.259	1.7	0.247
IP-CC2^124^	–0.592	0.592	0.695	6.1	0.347
IP-ADC(2)^124^	–0.529	0.529	0.655	5.5	0.369
IP-SCS-ADC(2)^124^	–0.299	0.345	0.436	3.6	0.304
IP-SOS-ADC(2)^124^	–0.182	0.288	0.353	3.0	0.289
IP-CCSD(T)^125^	–0.007	0.147	0.249	1.5	0.238

a Assessed based on the IPs calculated using various methods: IP-pCCD, IP-EOM-fpCCD, IP-EOM-fpCCSD, IP-EOM-fpLCCD, IP-EOM-fpLCCSD, IP-EOM-CCD(pCCD), and IP-EOM-CCSD(pCCD), listed in the upper part of each block, are derived in this work. All these CC approaches exploit natural pCCD-optimized orbitals (or the orbital-optimized pCCD reference determinant) and are conducted in the space of two-hole-one-particle (2h1p) states. The corresponding errors in IPs for CCSD done with Hartree-Fock orbitals, different variants of algebraic-diagrammatic construction (ADC(2), SCS-ADC(2), and SOS-ADC(2)) methods, and an approximate coupled cluster singles and doubles model (CC2) are presented in the lower part of each block. The errors in IPs were calculated concerning IP-EOM-CCSD(T)/aug-cc-pVDZ^125^ (top) and experimental data^126^ (bottom). The errors w.r.t. experimental values were calculated using 21 IPs, while those w.r.t. CCSD(T) were determined with 24 IPs. The definitions for ME, MAE, RMSE, MPE, and SD are ME = , MAE = , RMSE = , MPE = , SD =  - standard deviation of the mean signed errors.

**Figure 4:**
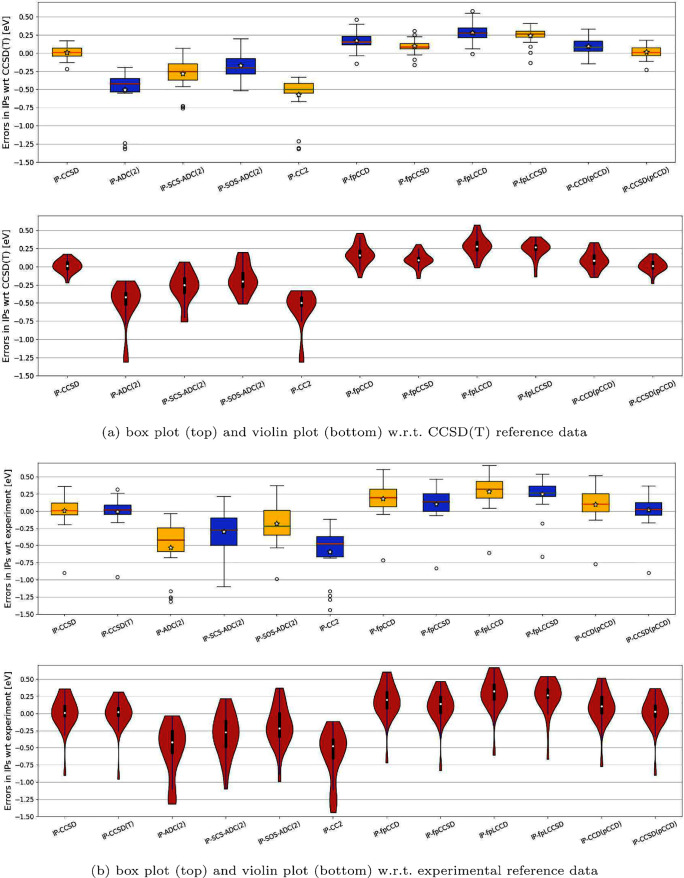
Box plots presented at the top and violin plots at the bottom, illustrating errors [eV] derived from selected methods (refer to Table 2 for numerical values). All errors are reported relative to either (a) IP-EOM-CCSD(T) or (b) experimental reference data. For brevity, we have omitted the EOM prefix in IP-EOM-CC-type methods. A star in each box plot denotes the mean value, while a white dot in each violin plot represents the median value.

Figure 4 displays the distribution of the errors in the organic acceptors’ IPs using box and violin plots. The errors in IPs calculated for the pCCD-based variants are shifted upward relative to the CCSD(T) reference. The mean and median values are located around the center of the box, indicating a rather symmetric distribution of the interquartile range. When outliers are excluded, the overall range is symmetric, suggesting a skewness close to zero. The corresponding violin plots (bottom) highlight that most of the IP error values are clustered around the median. Furthermore, the CCD variants exhibit a symmetric distribution, while the distribution of the CCSD-type corrections shows a slight tendency toward left-shifted skewness due to the presence of outliers. For experimental reference data, the distribution of the errors becomes wider and shifts upward. The smallest spread of errors is observed for IP-EOM-fpLCCSD, similar to IP-EOM-CCSD, but a larger error in the mean/median. In general (both with respect to experiment and CCSD(T) reference data), the addition of nonlinear broken-pair terms in IP-EOM-fpCCSD shifts the distribution closer to the reference but also increases the distribution’s width. Finally, the errors from the investigated pCCD-based methods feature a more compact and systematic distribution compared to those obtained from CC2 and different variants of ACD(2). Specifically, the skewness and locality of the errors predicted by pCCD-based methods are comparable to those of the discussed ADC(2) flavors, though the latter exhibit a larger spread of errors.

The error measures for our third and last test set, which contains the five nucleobases, are presented in Table 3, which summarizes errors in IPs extrapolated to the CBS limit. Most importantly, we observe the same trends as for the first two data sets. We should stress that all pCCD-based error measures are reduced by a factor of 2 when the experimental data^131^ are corrected for the zero-point energy (ZPE) and geometry relaxation effects as discussed in ref (127). For this particular reference set, the IP errors of pCCD-based methods are slightly higher in comparison to SCS-ADC(2) and SOS-ADC(2), ranging between 1.7% and 4.3% with respect to the corrected experimental values, while the ADC(2) variants predict errors between 0.9%–1.4%. For the uncorrected experimental data, the pCCD-based IP models provide the largest errors with an MPE of 4.3% to 7.0%.

**Table 3:** Statistical Error Measures [eV], Such as Mean Error (ME), Mean Absolute Error (MAE), Root-Mean-Square Error (RMSE), and Mean Percentage Error (MPE) Calculated for the Third Set of Molecules (Figure 2(c)—Nucleobases)^a^

	Errors w.r.t. experiment			
Method	ME	MAE	RMSE	MPE
IP-pCCD	–2.018	2.018	2.027	23.7
IP-fpCCD	0.497	0.497	0.505	5.8
IP-fpLCCD	0.600	0.600	0.605	7.0
IP-CCD(pCCD)	0.442	0.442	0.450	5.2
IP-fpCCSD	0.372	0.372	0.379	4.3
IP-fpLCCSD	0.508	0.508	0.514	6.0
IP-CCSD(pCCD)	0.310	0.310	0.321	3.6
IP-CCSD(HF)^124^	0.304	0.304	0.317	3.5
IP-CC2^124^	–0.218	0.218	0.235	2.6
IP-ADC(2)^124^	–0.124	0.124	0.166	1.5
SCS-ADC(2)^124^	0.097	0.138	0.161	1.6
SOS-ADC(2)^124^	0.208	0.208	0.250	2.4

	Errors w.r.t. experiment (corrected for zero-point energy and geometry relaxation)^b^			
Method	ME	MAE	RMSE	MPE
IP-pCCD	–2.238	2.238	2.244	25.6
IP-fpCCD	0.277	0.277	0.281	3.1
IP-fpLCCD	0.380	0.380	0.382	4.3
IP-CCD(pCCD)	0.222	0.222	0.226	2.5
IP-fpCCSD	0.152	0.152	0.157	1.7
IP-fpLCCSD	0.288	0.288	0.293	3.3
IP-CCSD(pCCD)	0.090	0.090	0.102	1.0
IP-CCSD(HF)^124^	0.084	0.090	0.100	1.0
IP-CC2^124^	–0.438	0.438	0.441	5.0
IP-ADC(2)^124^	–0.344	0.344	0.350	3.9
SCS-ADC(2)^124^	–0.123	0.123	0.149	1.4
SOS-ADC(2)^124^	–0.012	0.083	0.096	0.9

a Assessed based on the IPs calculated using various methods: IP-pCCD, IP-EOM-fpCCD, IP-EOM-fpCCSD, IP-EOM-fpLCCD, IP-EOM-fpLCCSD, IP-EOM-CCD(pCCD), and IP-EOM-CCSD(pCCD) with respect to the experimental values.^131^ Those IPs were extrapolated to the complete basis set (CBS) limit from the cc-pVDZ and cc-pVTZ data. All fpCC approaches exploit natural pCCD-optimized orbitals (or the orbital-optimized pCCD reference determinant) and are conducted in the space of two-hole-one-particle (2h1p) states. The definitions for ME, MAE, RMSE, and MPE are ME = , MAE = , RMSE = , MPE = .

b The corrected IP values provided in ref (127) of the reference experimental values provided in ref (131).

## 5 Conclusions

As recently shown,^74^ restricting the CC ansatz to the seniority-zero sector is insufficient in predicting reliable and accurate IPs. Although the seniority-zero pCCD model can capture static correlation reliably, it is inadequate to describe electron detachment with sufficient accuracy. This deficiency was attributed to the missing broken-pair states, that is the exclusion of the seniory-two, seniority-four, etc. sectors. In this work, we investigated the impact of dynamical correlation and the choice of the molecular orbital basis (canonical vs localized) on vertical ionization potentials using various pCCD-based and conventional CC approaches. Specifically, we studied six CC variants: IP-EOM-fpCCD, IP-EOM-fpLCCD, IP-EOM-CCD(pCCD), IP-EOM-fpCCSD, IP-EOM-fpLCCSD, and IP-EOM-CCSD(pCCD). Throughout this work, we included (up to) 2p1h operators in the IP-EOM formalism as the resulting pCCD-based model turned out to be superior to the corresponding IP-EOM approach restricted to 1h operators.^74^ Our analysis encompasses three sets of 70 molecules in total, comprising small organic molecules, medium-sized organic acceptors, and nucleobases, targeting 230 ionized states. These ionization energies are compared to IP-EOM-CCSD(T), IP-EOM-CCSDT, and experimental reference data. Furthermore, our results are juxtaposed with those obtained using various conventional CC methods, UCC flavors, and non-Dyson ADC second and third-order schemes.

Our statistical analysis (mean errors, mean absolute errors, root-mean-square errors, mean percentage errors, and standard deviations) highlights that all investigated frozen-pair coupled cluster methods feature similar performance. Specifically, the differences in errors are typically within chemical accuracy (1 kcal/mol or 0.05 eV), resulting in a mean percentage error between 1 to 3%. Adding single excitations slightly reduces error measures with respect to the corresponding CCD model. Yet, these changes approach chemical accuracy, constituting approximately 0.06 eV. Our benchmark data renders IP-EOM-fpCCSD the best-performing method among all tested frozen-pair variants. However, an in-depth analysis of box and violin plots suggests that decoupling the seniority-zero (pCCD) and seniority-two/four sectors (post-pCCD) deteriorates IPs compared to their composite optimization (CCD/CCSD). Specifically, while an LCC correction on top of pCCD may slightly reduce the width in IP errors, the corresponding median and distribution are shifted upward. The addition of nonlinear broken-pair terms in the fpCC counterpart brings the distribution closer to the reference, pointing to keep the seniority-sectors coupled during the optimization. Furthermore, the scattering of errors and their distribution around the median make frozen-pair methods comparable to the conventional IP-EOM-CCSD method. Noteworthy, the error measures of IP-EOM-fpCCSD are comparable to the accuracy of IP-EOM-UCC(3), identified as the best among recently investigated approximations.^117^ In general, the computationally cheaper IP-EOM-ADC(2) and IP-EOM-UCC2 flavors underestimate IPs and feature broader error distributions than the investigated IP-EOM-fpCC models. In contrast, the computationally more expensive IP-EOM-ADC(3) and IP-EOM-UCC3 variants (but similar in cost as IP-EOM-fpCC) overestimate IPs and predict considerably broader and more asymmetric error distributions compared to the IP-EOM-fpCC family of approaches. Finally, the influence of the molecular orbital basis or CC reference determinant (that is canonical vs localized) is marginal as the conventional IP-EOM-CCSD and IP-EOM-CCSD(pCCD) result in almost identical errors in ionization potentials. These observations suggest that triple excitations are crucial for further improving IPs and approaching chemical accuracy for modeling electron detachment processes with pCCD-based methods.

## Data Availability

The data that support the findings of this study are available within the article and its Supporting Information and from the corresponding author upon reasonable request.

## Supporting Information Available

Vertical ionization energies calculated with the IP-EOM-fpCCD, IP-EOM-fpCCSD, IP-EOM-fpLCCD, IP-EOM-fpLCCSD, IP-EOM-CCD(pCCD), and IP-EOM-CCSD(pCCD) methods (PDF)
