# Supplementary material for: Benchmarking Ionization Potentials from pCCD Tailored Coupled Cluster Models
Source: J Chem Theory Comput. 2024 May 16;20(10):4182–95. doi: 10.1021/acs.jctc.4c00172 (PMC11137826; doi:10.1021/acs.jctc.4c00172)
Supplement: Supplementary file 1 — ct4c00172_si_001.pdf [file ct4c00172_si_001.pdf]

Benchmarking ionization potentials from pCCD tailored coupled cluster models

Marta Gałyńska and Katharina Boguslawski*

Institute of Physics, Faculty of Physics, Astronomy, and Informatics, Nicolaus Copernicus University in Toruń, Grudziadzka 5, 87-100 Toruń, Poland

*E-mail: k.boguslawski@fizyka.umk.pl

Supplementary Information

S1 General remarks

Table S1: Ionization potentials of doublet states for which at least EOM-IP-CCSDT reference data are available in Ref. [1] computed with various IP-EOM-pCCD schemes. All IPs are given in units of eV. We have dropped the label "IP-EOM" for each CC approach. All listed IPs were computed using the cc-pVTZ basis set and employing geometries optimized at the CCSD(T)/aug-cc-pVTZ level of theory, as provided in the supplementary information of Refs. [1,6].

Compound	Irrep.	Exp.	CCSDT	fpCCD	fpLCCD	CCD(pCCD)	fpCCSD	fpLCCSD	CCSD(pCCD)
P_2	$2\pi_u$	10.65	10.51	10.74	10.82	10.62	10.69	10.78	10.57
	$5\sigma_g$	10.84	10.65	10.78	10.86	10.83	10.69	10.76	10.74
SiF_4	$1t_1$	16.40	16.23	16.40	16.42	16.36	16.42	16.46	16.37
	$5t_2$	17.50	17.24	17.41	17.43	17.37	17.45	17.48	17.40
	$1e$	18.10	17.63	17.82	17.84	17.78	17.85	17.89	17.81
	$4t_2$	19.50	19.17	19.37	19.39	19.33	19.41	19.44	19.36
	$5a_1$	21.55	21.27	21.49	21.51	21.46	21.54	21.58	21.50
CH_4	$1t_2$	13.60	14.35	14.46	14.47	14.45	14.40	14.41	14.38
	$2a'$	22.90	23.11	23.48	23.49	23.47	23.39	23.41	23.38
OCS	2π	11.24	11.12	11.26	11.33	11.24	11.21	11.31	11.20
	1π	15.53	15.61	16.42	16.49	16.35	16.27	16.33	16.14
	4σ	16.04	15.83	16.17	16.23	16.19	16.05	16.12	16.08
	3σ	17.96	17.89	18.63	18.70	18.59	18.54	18.60	18.43
HCOOH	$10a'$	11.50	11.33	11.50	11.51	11.57	11.50	11.54	11.45
	$2a''$	12.60	12.47	12.63	12.60	12.68	12.63	12.67	12.55
	$9a'$	14.80	14.79	14.98	14.95	15.01	14.98	15.01	14.94
	$1a''$	15.80	15.71	16.13	16.10	16.16	16.13	16.17	16.05
	$8a'$	17.10	17.06	17.38	17.39	17.45	17.38	17.42	17.33
	$7a'$	17.80	17.81	18.28	18.20	18.24	18.28	18.31	18.24
	$6a'$	22.00	22.08	22.67	22.58	22.62	22.67	22.70	22.63
NH_3	$3a_1$	10.80	10.74	10.80	10.82	10.79	10.74	10.77	10.72
	$1e$	16.00	16.48	16.56	16.58	16.53	16.53	16.55	16.48
C_2H_2	$1\pi_u$	11.49	11.42	11.71	11.76	11.59	11.63	11.69	11.52
	$3\sigma_g$	16.70	17.12	17.37	17.41	17.33	17.26	17.29	17.22
	$2\sigma_u$	18.70	19.00	19.29	19.32	19.23	19.19	19.22	19.14
	$2\sigma_g$	23.50	23.70	24.56	24.60	24.54	24.44	24.48	24.42
C_2H_4	$1b_{3u}$	10.68	10.64	10.87	10.91	10.73	10.78	10.83	10.65
	$1b_{3g}$	12.80	13.08	13.23	13.25	13.19	13.15	13.18	13.12
	$3a_g$	14.80	14.79	15.01	15.04	14.99	14.90	14.94	14.88
	$1b_{2u}$	16.00	16.14	16.40	16.43	16.35	16.35	16.38	16.30
	$2b_{1u}$	19.10	19.32	19.74	19.77	19.68	19.66	19.69	19.61
	$2a_g$	23.60	23.87	24.61	24.64	24.55	24.49	24.52	24.44
CH_3CN	$2e$	12.46	12.45	12.82	12.88	12.70	12.72	12.78	12.59
	$7a_1$	13.17	12.97	13.28	13.32	13.22	13.17	13.23	13.12
	$1e$	15.70	16.24	16.50	16.52	16.47	16.41	16.43	16.38
	$6a_1$	17.40	17.38	17.71	17.75	17.68	17.56	17.59	17.54
	$5a_1$	24.90	24.88	25.52	25.56	25.48	25.39	25.44	25.35
N_2	$3\sigma_g$	15.58	15.45	15.70	15.73	15.68	15.56	15.59	15.55
	$1\pi_u$	16.93	16.89	17.41	17.47	17.24	17.32	17.38	17.15
	$2\sigma_u$	18.75	18.67	18.96	19.00	18.93	18.85	18.89	18.83
SiO	7σ	11.61	11.33	11.39	11.42	11.41	11.45	11.52	11.45
	2π	12.19	11.87	11.84	11.88	11.75	12.03	12.19	11.92
	6σ	14.80	14.58	14.97	15.00	14.98	15.06	15.16	15.05

Table S1: Ionization potentials of doublet states for which at least EOM-IP-CCSDT reference data are available in Ref. [1] computed with various IP-EOM-pCCD schemes. All IPs are given in units of eV. We have dropped the label "IP-EOM" for each CC approach. All listed IPs were computed using the cc-pVTZ basis set and employing geometries optimized at the CCSD(T)/aug-cc-pVTZ level of theory, as provided in the supplementary information of Refs. [1,6].

Compound	Irrep.	Exp.	CCSDT	fpCCD	fpLCCD	CCD(pCCD)	fpCCSD	fpLCCSD	CCSD(pCCD)
CH_3CCH	$2e$	10.54	10.46	10.76	10.81	10.66	10.66	10.72	10.56
	$1e$	14.60	15.17	15.37	15.39	15.34	15.30	15.33	15.27
	$7a_1$	15.40	15.22	15.51	15.55	15.47	15.36	15.41	15.33
	$6a_1$	17.40	17.65	18.04	18.07	17.99	17.92	17.96	17.86
H_2O	$1b_2$	18.55	18.84	18.85	18.87	18.80	18.86	18.89	18.80
	$3a_1$	14.74	14.69	14.66	14.68	14.63	14.66	14.70	14.63
	$1b_1$	12.62	12.45	12.45	12.47	12.40	12.45	12.48	12.39
C_2H_6	$1e_g$	12.00	12.68	12.80	12.81	12.78	12.72	12.75	12.70
	$3b_{1g}$	12.70	13.06	13.24	13.27	13.22	13.13	13.16	13.10
	$2e_u$	15.00	15.38	15.51	15.52	15.49	15.45	15.47	15.43
	$2b_u$	20.40	20.69	21.02	21.03	21.00	20.94	20.96	20.92
	$2a_{1g}$	23.90	24.15	24.79	24.78	24.74	24.65	24.67	24.63
C_3O_2	$2\pi_u$	10.80	10.62	10.78	10.88	10.77	10.67	10.81	10.68
	$1\pi_g$	15.00	14.92	15.71	15.80	15.64	15.65	15.78	15.93
	$1\pi_u$	16.00	15.72	16.49	16.57	16.45	16.35	16.42	16.31
	$5\sigma_u$	17.30	17.10	17.78	17.86	17.73	17.73	17.83	17.66
	$6\sigma_g$	17.80	17.28	17.98	18.06	17.93	17.95	18.06	17.87
	$4\sigma_u$	21.90	22.22	23.20	23.26	23.18	22.88	22.92	22.89
	$5\sigma_g$	25.60	25.88	25.78	25.90	25.76	25.65	25.78	25.65
C_2F_4	$2b_{1u}$	10.69	10.37	10.63	10.71	10.65	10.49	10.56	10.49
	$4b_{1g}$	15.90	15.78	15.98	16.02	15.95	16.00	16.06	15.96
	$6a_g$	16.60	16.11	16.38	16.41	16.34	16.34	16.39	16.14
	$4b_{2u}$	16.60	16.32	16.53	16.57	16.49	16.57	16.62	16.52
	$1a_u$	16.60	16.48	16.72	16.76	16.69	16.73	16.78	16.69
	$1b_{3g}$	16.60	16.64	16.90	16.93	16.87	16.92	16.96	16.87
	$5b_{3u}$	17.60	17.32	17.57	17.60	17.53	17.60	17.65	17.55
	$1b_{2g}$	18.20	18.02	18.24	18.27	18.20	18.29	18.36	18.25
	$1b_{1u}$	19.40	19.12	19.56	19.60	19.55	19.51	19.55	19.49
	$3b_{1g}$	19.40	19.41	19.71	19.75	19.65	19.69	19.75	19.62
	$3b_{2u}$	21.00	20.76	21.03	21.07	20.97	21.04	21.10	20.97
	$5a_g$	21.00	21.03	21.39	21.43	21.35	21.36	21.41	21.31
C_2N_2	$1\pi_g$	13.51	13.48	13.94	14.03	13.80	13.82	13.92	13.68
	$5\sigma_g$	14.49	14.40	14.81	14.87	14.76	14.67	14.74	14.63
	$4\sigma_u$	14.86	14.77	15.17	15.24	15.12	15.06	15.13	15.02
	$1\pi_u$	15.60	15.70	16.28	16.35	16.15	16.15	16.20	16.02
	$4\sigma_g$	22.80	23.29	24.21	24.27	24.15	23.96	24.00	23.92
NSF	$13a'$	11.82	11.62	11.85	11.92	11.83	11.75	11.87	11.74
	$12a'$	13.50	13.30	13.63	13.69	13.61	13.52	13.60	13.51
	$3a''$	13.87	13.74	14.07	14.14	13.95	14.05	14.16	13.92
	$11a'$	15.62	15.24	15.59	15.64	15.55	15.61	15.68	15.57
	$2a''$	16.47	16.11	16.57	16.62	16.48	16.56	16.62	16.47
	$10a'$	17.20	16.35	16.90	16.96	16.89	16.88	16.95	16.86
NNO	2π	12.89	12.74	12.82	12.91	12.75	12.85	13.05	12.80
	7σ	16.38	16.28	17.03	17.09	16.99	16.72	16.71	16.65
	1π	18.23	18.31	19.27	19.33	19.18	19.07	19.11	18.97
	6σ	20.11	19.89	20.04	20.10	19.99	20.12	20.30	20.12
HCCCN	2π	11.75	11.69	12.06	12.15	11.95	11.95	12.05	11.84

Table S1: Ionization potentials of doublet states for which at least EOM-IP-CCSDT reference data are available in Ref. [1] computed with various IP-EOM-pCCD schemes. All IPs are given in units of eV. We have dropped the label "IP-EOM" for each CC approach. All listed IPs were computed using the cc-pVTZ basis set and employing geometries optimized at the CCSD(T)/aug-cc-pVTZ level of theory, as provided in the supplementary information of Refs. [1,6].

Compound	Irrep.	Exp.	CCSDT	fpCCD	fpLCCD	CCD(pCCD)	fpCCSD	fpLCCSD	CCSD(pCCD)
	9σ	13.54	13.44	13.85	13.91	13.79	13.74	13.82	13.68
	1π	14.18	14.19	14.70	14.77	14.58	14.58	14.64	14.46
	8σ	18.30	18.53	18.96	19.01	18.91	18.80	18.84	18.77
	7σ	21.30	21.55	22.19	22.25	22.13	21.98	22.03	21.94
	6σ	25.00	25.31	26.04	26.10	26.01	25.88	25.93	25.86
O_3	$6a_1$	12.73	12.54	13.03	13.09	12.83	12.93	13.02	12.76
	$4b_2$	13.00	12.69	13.29	13.34	13.05	13.19	13.25	12.93
	$1a_2$	13.54	13.46	14.59	14.67	13.78	14.49	14.57	13.55
	$1b_1$	19.99	21.32	18.37	18.46	18.43	18.28	18.43	18.56
CO	5σ	14.01	13.90	14.19	14.22	14.27	14.02	14.04	14.13
	1π	16.91	16.95	17.12	17.16	16.99	17.16	17.23	17.00
	4σ	19.72	19.54	19.77	19.81	19.72	19.85	19.91	19.76
HF	1π	16.19	15.90	15.81	15.83	15.76	15.88	15.90	15.81
	3σ	19.90	19.83	19.75	19.77	19.70	19.82	19.84	19.75
CS	7σ	11.34	11.26	11.66	11.71	11.65	11.44	11.49	11.46
	2π	12.90	12.88	13.11	13.18	13.01	13.07	13.15	12.97
	6σ	18.03	18.08	17.21	17.27	17.22	17.15	17.22	17.16
F_2	$1\pi_g$	15.87	15.58	15.62	15.65	15.48	15.63	15.67	15.51
	$1\pi_u$	18.80	18.74	18.98	19.01	18.79	19.00	19.03	18.82
	$3\sigma_g$	21.10	20.96	21.45	21.49	21.00	21.48	21.53	21.05
HCN	1π	13.61	13.72	14.08	14.14	13.93	14.01	13.93	13.87
	5σ	14.01	13.84	14.04	14.08	13.99	13.96	13.99	13.91
	4σ	19.86	20.43	20.78	20.81	20.74	20.65	20.74	20.62
CH_3F	$2e$	13.10	13.17	13.21	13.23	13.18	13.22	13.25	13.18
	$5a_1$	17.00	17.01	17.09	17.12	17.03	17.11	17.16	17.04
	$1e$	17.00	17.04	17.17	17.19	17.15	17.17	17.20	17.14
	$4a_1$	23.40	23.51	23.90	23.92	23.89	23.83	23.85	23.81
HCl	2π	12.77	12.58	12.69	12.73	12.66	12.66	12.69	12.63
	5σ	16.60	16.57	16.68	16.71	16.67	16.65	16.68	16.64
	4σ	25.80	26.63	25.75	25.79	25.73	25.71	25.75	25.70
CO_2	$1\pi_g$	13.79	13.63	13.76	13.82	13.73	13.72	13.82	13.69
	$1\pi_u$	17.60	17.50	18.05	18.10	18.02	17.97	18.04	17.93
	$3\sigma_u$	18.08	17.84	18.16	18.21	18.14	18.11	18.18	18.08
	$4\sigma_g$	19.40	19.08	19.47	19.52	19.45	19.44	19.52	19.42
cis$-C_2H_2F_2$	$2b_1$	10.62	10.39	10.67	10.72	10.57	10.57	10.62	10.46
	$7a_1$	14.00	13.88	14.05	14.08	14.01	14.04	14.08	14.00
	$6b_2$	14.90	14.80	14.94	14.97	14.89	14.98	15.03	14.93
	$1a_2$	16.20	16.07	16.17	16.20	16.11	16.27	16.34	16.21
	$5b_2$	17.10	16.85	17.16	17.20	17.11	17.15	17.20	17.10
	$1b_1$	17.10	16.97	17.12	17.14	17.05	17.15	17.19	17.07
	$6a_1$	18.80	18.50	18.76	18.80	18.69	18.76	18.82	18.70
	$5a_1$	18.80	18.88	19.22	19.26	19.18	19.20	19.25	19.15
	$4b_2$	20.90	20.85	21.28	21.31	21.21	21.26	21.30	21.19
	$4a_1$	25.20	24.61	26.33	26.39	26.26	25.09	25.17	25.04
gem$-C_2H_2F_2$	$2b_1$	10.70	10.54	10.85	10.90	10.75	10.73	10.78	10.63
	$5b_2$	14.90	14.96	15.15	15.18	15.11	15.13	15.17	15.09
	$8a_1$	15.80	15.52	15.72	15.76	15.68	15.70	15.75	15.66

Table S1: Ionization potentials of doublet states for which at least EOM-IP-CCSDT reference data are available in Ref. [1] computed with various IP-EOM-pCCD schemes. All IPs are given in units of eV. We have dropped the label "IP-EOM" for each CC approach. All listed IPs were computed using the cc-pVTZ basis set and employing geometries optimized at the CCSD(T)/aug-cc-pVTZ level of theory, as provided in the supplementary information of Refs. [1,6].

Compound	Irrep.	Exp.	CCSDT	fpCCD	fpLCCD	CCD(pCCD)	fpCCSD	fpLCCSD	CCSD(pCCD)
	$4b_2$	16.10	15.85	16.05	16.08	16.01	16.04	16.09	16.00
	$1a_2$	16.10	16.02	16.17	16.20	16.11	16.25	16.32	16.19
	$7a_1$	18.20	18.27	18.35	18.38	18.29	18.42	18.49	18.36
	$1b_1$	18.20	18.10	18.43	18.46	18.39	18.38	18.42	18.34
	$3b_2$	19.70	19.63	19.87	19.91	19.80	19.92	20.01	19.86
	$6a_1$	21.50	21.41	21.83	21.86	21.76	21.85	21.92	21.79
	$5a_1$	25.20	25.10	25.73	25.77	25.69	25.61	25.66	25.57
FCN	2π	13.65	13.50	13.90	13.96	13.78	13.80	13.86	13.68
	7σ	14.56	14.31	14.60	14.64	14.55	14.48	14.54	14.44
	1π	19.30	19.39	19.67	19.71	19.61	19.69	19.74	19.64
	6σ	22.60	22.79	23.09	23.13	23.03	23.09	23.16	23.05
H_2CO	$2b_2$	10.90	10.75	10.85	10.88	10.79	10.85	10.90	10.77
	$1b_1$	14.50	14.50	14.74	14.78	14.56	14.74	14.81	14.55
	$5a_1$	16.10	15.96	16.11	16.15	16.05	16.13	16.19	16.05
	$1b_2$	17.00	17.10	17.52	17.54	17.47	17.46	17.49	17.43
	$4a_1$	21.40	21.30	21.84	21.86	21.80	21.74	21.76	21.72
H_2CS	$3b_2$	9.38	9.25	9.34	9.38	9.33	9.31	9.36	9.29
	$2b_1$	11.76	11.78	12.07	12.12	11.90	12.00	12.07	11.84
	$7a_1$	13.85	13.88	14.06	14.11	14.06	13.99	14.05	13.99
	$2b_2$	15.20	15.61	16.11	16.15	16.04	16.00	16.03	15.95
	$6a_1$	19.90	19.60	19.61	19.66	19.55	19.53	19.57	19.47
HCCF	2π	11.50	11.34	11.60	11.66	11.57	11.50	11.57	11.47
	1π	18.00	17.90	18.04	18.08	18.01	18.13	18.20	18.09
	7σ	18.00	18.12	18.43	18.47	18.43	18.29	18.31	18.28
	6σ	21.20	20.95	21.23	21.27	21.19	21.27	21.36	21.24
	5σ	24.30	24.40	25.46	25.50	25.44	25.32	25.35	25.29
$HCONH_2$	$10a'$	10.40	10.17	10.41	10.45	10.36	10.41	10.47	10.32
	$2a''$	10.70	10.56	10.70	10.75	10.65	10.65	10.73	10.60
	$1a''$	14.10	14.06	14.58	14.62	14.50	14.52	14.57	14.44
	$9a'$	14.80	14.67	15.00	15.05	14.95	14.99	15.04	14.90
	$8a'$	16.30	16.55	17.01	17.04	16.98	16.92	16.97	16.89
	$7a'$	18.80	19.25	19.11	19.14	19.07	19.05	19.11	19.03
	$6a'$	20.70	20.90	21.40	21.43	21.37	21.28	21.31	21.26
HC_4H	$1\pi_g$	10.30	10.20	10.52	10.62	10.42	10.42	10.52	10.32
	$1\pi_u$	12.71	12.70	13.13	13.19	13.03	13.02	13.07	12.92
	$5\sigma_g$	17.00	17.14	17.55	17.61	17.50	17.39	17.44	17.35
	$4\sigma_u$	17.50	17.82	18.25	18.31	18.20	18.12	18.17	18.08
	$4\sigma_g$	20.00	20.05	20.63	20.69	20.56	20.46	20.51	20.40
	$3\sigma_u$	23.30	23.42	24.22	24.28	24.18	24.07	24.12	24.03
	$3\sigma_g$	25.00	24.76	25.54	25.60	25.50	25.39	25.45	25.36
CH_2F_2	$2b_1$	13.30	13.29	13.39	13.41	13.37	13.37	13.40	13.34
	$4b_2$	15.40	14.92	14.94	14.97	14.90	15.00	15.05	14.95
	$6a_1$	15.40	15.20	15.28	15.31	15.24	15.30	15.34	15.25
	$1a_2$	15.80	15.59	15.65	15.67	15.61	15.71	15.76	15.66
	$3b_2$	19.10	18.75	18.89	18.92	18.82	18.92	18.98	18.85
	$5a_1$	19.10	18.97	19.16	19.19	19.12	19.18	19.22	19.13
	$1b_1$	19.10	19.15	19.37	19.39	19.36	19.35	19.38	19.33

Table S1: Ionization potentials of doublet states for which at least EOM-IP-CCSDT reference data are available in Ref. [1] computed with various IP-EOM-pCCD schemes. All IPs are given in units of eV. We have dropped the label "IP-EOM" for each CC approach. All listed IPs were computed using the cc-pVTZ basis set and employing geometries optimized at the CCSD(T)/aug-cc-pVTZ level of theory, as provided in the supplementary information of Refs. [1,6].

**Compound**	**Irrep.**	**Exp.**	**CCSDT**	**fpCCD**	**fpLCCD**	**CCD(pCCD)**	**fpCCSD**	**fpLCCSD**	**CCSD(pCCD)**
CF_4	$1t_1$	16.20	16.09	16.26	16.29	16.22	16.25	16.29	16.20
	$4t_2$	17.40	17.24	17.41	17.44	17.36	17.41	17.46	17.36
	$1e$	18.50	18.18	18.41	18.43	18.37	18.40	18.44	18.36
	$3t_2$	22.10	21.93	22.23	22.26	22.18	22.23	22.28	22.17
	$4a_1$	25.10	24.85	25.25	25.28	25.19	25.23	25.27	25.17
CHF_3	$6a_1$	14.80	14.76	14.92	14.95	14.90	14.90	14.93	14.86
	$1a_2$	15.50	15.33	15.42	15.45	15.38	15.45	15.49	15.40
	$5e$	16.20	15.93	16.03	16.05	15.98	16.06	16.11	16.01
	$4e$	17.20	16.99	17.13	17.16	17.09	17.16	17.20	17.11
	$3e$	20.70	20.41	20.63	20.66	20.57	20.65	20.70	20.58
	$5a_1$	20.70	20.93	21.23	21.25	21.20	21.21	21.24	21.17
	$4a_1$	24.40	24.43	24.83	24.85	24.79	24.79	24.83	24.75
CH_3NC	$7a_1$	11.32	11.21	11.57	11.61	11.61	11.38	11.41	11.44
	$2e$	12.50	12.55	12.78	12.83	12.69	12.74	12.81	12.63
	$1e$	16.10	16.59	16.75	16.78	16.72	16.74	16.78	16.68
	$6a_1$	18.20	18.38	18.72	18.76	18.68	18.68	18.73	18.62
	$5a_1$	25.00	25.06	25.59	25.63	25.56	25.54	25.59	25.49

Table S2: Ionization potentials of doublet states computed with various IP-EOM-pCCD schemes using the aug-cc-pVDZ basis set. IPs calculated with CCSD(T)/aug-cc-pVDZ are obtained from Ref. [7], the experimental values are taken from Ref. [4]. All IPs are given in units of eV. We have dropped the label "IP-EOM" for each CC approach. The molecular structures relaxed at the B3LYP/6-311G** were obtained from the suplementary materials of Ref. [2].

**Compound**	**pCCD**	**fpCCD**	**fpCCSD**	**fpLCCD**	**fpLCCSD**	**CCD(pCCD)**	**CCSD(pCCD)**	**CCSD(T)**	**Exp**
Acridine	5.69	8.01	7.94	8.15	8.14	7.90	7.82	7.87	7.8
Anthracene	5.18	7.49	7.42	7.64	7.67	7.39	7.31	7.37	7.44
Azulene	5.10	7.38	7.36	7.52	7.24	7.29	7.26	7.38	7.42
Benzonitrile	7.66	9.84	9.80	9.96	10.00	9.77	9.73	9.76	9.73
Benzoquinone	8.18	10.14	10.13	10.19	10.21	10.07	10.06	10.02	10.0
Dichlone	7.80	9.95	9.85	10.04	9.96	9.87	9.76	9.76	9.5
Fumaronitrile	9.69	11.58	11.48	11.66	11.56	11.45	11.35	11.25	11.3
Maleic anhydride	9.05	11.07	11.12	11.11	11.20	11.00	11.05	11.05	11.07
mDCNB	8.17	10.40	10.34	10.52	10.53	10.32	10.26	10.24	10.20
Napthalenedione	7.60	9.82	9.79	9.94	10.03	9.75	9.72	9.65	9.5
NDCA	6.88	9.15	9.04	9.28	9.20	9.06	8.94	8.96	8.92
Nitrobenzene	7.98	10.15	10.10	10.26	10.30	10.09	10.04	10.03	9.94
Nitrobenzonitrile	8.40	10.65	10.56	10.77	10.74	10.58	10.49	10.42	10.59
Phenazine	6.16	8.46	8.40	8.61	8.54	8.36	8.27	8.31	8.44
Phthalic anhydrid	8.27	10.52	10.45	10.63	10.64	10.45	10.40	10.41	10.1
Phthalimide	7.86	10.05	9.96	10.16	10.14	9.99	9.91	9.91	9.90
TCNE	10.11	12.20	12.05	12.32	12.15	12.07	11.92	11.74	11.79
Cl4benzoquinone	8.55	10.34	10.21	10.41	10.27	10.26	10.11	9.99	9.74
Cl4isobenzofuranedione	7.93	10.08	9.97	10.19	10.13	10.03	9.90	9.84	10.8
F4benzenedicarbonitrile	8.43	10.73	10.65	10.85	10.91	10.67	10.59	10.52	10.65
F4benzoquinone	9.34	11.29	11.14	11.36	11.20	11.20	11.05	10.89	10.7
Bodipy	5.37	7.74	7.80	7.87	7.98	7.74	7.82	7.89	-
Dinitrobenzonitrile	9.01	11.28	11.15	11.40	11.31	11.20	11.08	11.31	-
TCNQ	7.48	9.73	9.56	9.90	9.72	9.63	9.45	9.35	-

Table S3: Ionization potentials of doublet states computed with various IP-EOM-pCCD schemes. The reference experimental energies are taken from Ref. [5] and their corrected values from Ref. [8]. All IPs are given in units of eV. We have dropped the label "IP-EOM" for each CC approach. The molecular geometries were optimized using the B3LYP/6-31G(2df,p) method (Refs. [8, 3]).

Compound	pCCD	fpCCD	fpCCSD	fpLCCD	fpLCCSD	CCD(pCCD)	CCSD(pCCD)	Exp	Exp-C[a]
Adenine									
cc-pVDZ	6.05	8.15	8.05	8.24	8.15	8.08	7.96		
cc-pVTZ	6.03	8.51	8.39	8.62	8.51	8.45	8.30		
CBS	6.02	8.65	8.53	8.78	8.67	8.60	8.45	8.26	8.45
Guanine									
cc-pVDZ	5.58	7.73	7.65	7.82	7.79	7.67	7.58		
cc-pVTZ	5.60	8.12	8.02	8.23	8.20	8.07	7.96		
CBS	5.60	8.28	8.18	8.40	8.37	8.23	8.12	7.77	7.99
Cytosine									
cc-pVDZ	6.51	8.51	8.44	8.58	8.53	8.44	8.36		
cc-pVTZ	6.55	8.92	8.82	9.00	8.93	8.85	8.75		
CBS	6.57	9.08	8.98	9.18	9.11	9.03	8.91	8.68	8.83
Uracil									
cc-pVDZ	7.48	9.37	9.24	9.42	9.32	9.29	9.17		
cc-pVTZ	7.50	9.73	9.59	9.81	9.69	9.67	9.53		
CBS	7.51	9.88	9.74	9.97	9.85	9.82	9.69	9.32	9.58
Thymine									
cc-pVDZ	7.08	8.99	8.86	9.04	8.94	8.91	8.79		
cc-pVTZ	7.10	9.34	9.19	9.42	9.30	9.27	9.13		
CBS	7.11	9.49	9.33	9.57	9.45	9.42	9.28	8.87	9.15

[a] the experimental data provided in Ref. [5] were corrected in Ref. [8] with zero-point energy and geometry relaxation.

References

[1] A. L. Dempwolff, M. Hodecker, and A. Dreuw. Vertical ionization potential benchmark for unitary coupled-cluster and algebraic-diagrammatic construction methods. *J. Chem. Phys.*, 156(5):054114, 2022.

[2] J. W. Knight, X. Wang, L. Gallandi, O. Dolgounitcheva, X. Ren, J. V. Ortiz, P. Rinke, T. Körzdörfer, and N. Marom. Accurate Ionization Potentials and Electron Affinities of Acceptor Molecules III: A Benchmark of GW Methods. *J. Chem. Theory Comput.*, 12(2):615–626, 2016.

[3] D. Kumar, A. K. Dutta, and P. U. Manohar. Resolution of the Identity and Cholesky Representation of EOM-MP2 Approximation: Implementation, Accuracy and Efficiency. *J. Chem. Sci.*, 129(10):1611–1626, 2017.

[4] S. G. Lias, J. E. Bartmess, J. F. Liebman, J. L. Holmes, R. D. Levin, and W. G. Mallard. *Ion Energetics Data in NIST Chemistry Webbook, NIST Standard Reference Database Number 69*. National Institute of Standards and Technology, 2015.

[5] V. Orlov, A. Smirnov, and Y. Varshavsky. Ionization potentials and electron-donor ability of nucleic acid bases and their analogues. *Tetrahedron Lett.*, 17(48):4377–4378, 1976.

[6] D. S. Ranasinghe, J. T. Margraf, A. Perera, and R. J. Bartlett. Vertical valence ionization potential benchmarks from equation-of-motion coupled cluster theory and QTP functionals. *J. Chem. Phys.*, 150(7):074108, 2019.

[7] R. M. Richard, M. S. Marshall, O. Dolgounitcheva, J. V. Ortiz, J.-L. Brédas, N. Marom, and C. D. Sherrill. Accurate Ionization Potentials and Electron Affinities of Acceptor Molecules I. Reference Data at the CCSD(T) Complete Basis Set Limit. *J. Chem. Theory Comput.*, 12(2):595–604, 2016.

[8] S. Śmiga, S. Siecińska, and I. Grabowski. From simple molecules to nanotubes. Reliable predictions of ionization potentials from the ΔMP2-SCS methods. *New J. Phys.*, 22(8):083084, 2020.
